# Bariatric Surgery Before Abdominoplasty Is Associated with Increased Perioperative Anemia, Hemoglobin Loss and Drainage Fluid Volume: Analysis of 505 Body Contouring Procedures

**DOI:** 10.3390/jcm14113783

**Published:** 2025-05-28

**Authors:** Tonatiuh Flores, Jana Schön, Christina Glisic, Kristina Pfoser, Celina Kerschbaumer, Martin S. Mayrl, Klaus F. Schrögendorfer, Konstantin D. Bergmeister

**Affiliations:** 1 Karl Landsteiner University of Health Sciences, Dr. Karl-Dorrek-Straße 30, 3500 Krems, Austria; jana.schoen2@stpoelten.lknoe.at (J.S.); christina.glisic@stpoelten.lknoe.at (C.G.); kristina.pfoser@stpoelten.lknoe.at (K.P.); celina.kerschbaumer@outlook.com (C.K.); martin.mayrl@gmx.at (M.S.M.); klaus.schroegendorfer@stpoelten.lknoe.at (K.F.S.); konstantin.bergmeister@stpoelten.lknoe.at (K.D.B.); 2 Division of Plastic, Aesthetic and Reconstructive Surgery, University Hospital St. Pölten, Dunant-Platz 1, 3100 St. Pölten, Austria; 3 Clinical Laboratory for Bionic Extremity Reconstruction, University Clinic for Plastic, Reconstructive and Aesthetic Surgery, Medical University of Vienna, 1090 Vienna, Austria

**Keywords:** bariatric surgery, body contouring, anemia

## Abstract

**Background**: The global rise in obesity rates has led to an increase in bariatric procedures, resulting in more patients needing relief from excess skin through body contouring surgeries (BCS), such as abdominoplasty. Although these procedures are generally considered safe, they can be associated with notable perioperative complications, including increased Hb-loss (hemoglobin loss) and drainage fluid volumes. Thus, this study analyzed risk factors for prolonged fluid secretion after abdominoplasty. **Methods**: We retrospectively analyzed 505 body contouring procedures performed between January 2018 and December 2023 at the Department of Plastic Surgery at the University Clinic of St. Poelten. The investigation focused on postoperative Hb-loss, drainage fluid volumes and hemoglobin levels in patients, comparing those with and without prior bariatric surgery. Statistical analyses utilized the *t*-test for Equality of Means, while correlation analyses were conducted using Spearman Rho and the Mann–Whitney U test. **Results**: Bariatric patients demonstrated markedly reduced hemoglobin levels both preoperatively (13.24 g/dL) and postoperatively (10.68 g/dL) compared to their non-bariatric counterparts (14.02 g/dL preoperatively and 11.71 g/dL postoperatively; *p* < 0.001). The prevalence of anemia was likewise substantially higher in the bariatric cohort, rising from 14.52% preoperatively to 82.48% postoperatively, versus 6.25% and 61.25%, respectively, in the non-bariatric group (*p* = 0.001). Moreover, prior bariatric surgery was significantly associated with increased postoperative drainage volume (*p* = 0.009) and prolonged operative time (*p* = 0.002). Notably, extended hospital stays exhibited a strong correlation with postoperative anemia (*p* = 0.005). **Conclusions**: Collectively, our findings underscore the potential risk of increased hemoglobin loss at BCS after bariatric weight loss. Tailored hemoglobin management and nutritional strategies are essential to improve the outcomes and safety of post-bariatric BCS. Meticulous preoperative identification of hematological discrepancies and adequate patient preparation are imperative for positive postoperative patient safety.

## 1. Introduction

Bariatric surgery is regarded as the gold standard for patients suffering from severe obesity (BMI > 40 kg/m^2^) [1,2,3,4]. Yet, while this intervention leads to significant and often life-changing weight loss, it also introduces new challenges. Most notably, patients frequently suffer from excess skin, which can limit mobility, cause dermatological issues and potentially lead to depression [5,6,7,8,9,10,11,12]. In response to these complications, plastic surgery becomes essential. Procedures such as body contouring surgery (BCS), and in particular abdominoplasty, play a crucial role in restoring body contours and enhancing patients’ self-image [4,9,10,13,14,15]. BCS is an essential aspect of the plastic surgical armamentarium. Through these interventions, patients who have experienced massive weight loss are better able to reintegrate into society and alleviate both their physical and psychological burdens [14,15,16,17,18].

Such procedures address a variety of problems for the affected individuals, namely mobility impairment, intertriginous skin conditions and psychological distress [1,2,3,4]. However, BCS is associated with numerous perioperative complications including increased Hb-loss, skin necrosis and infection [5,6,7]. These complications can prolong inpatient treatment and thus increase psychological stress and healthcare costs [9]. Despite these challenges, the value of BCS remains clear. For many patients, it offers crucial support after massive weight loss and represents the final step in their rehabilitation, enabling a return to normal life [2,3,4,13,14].

This paper examines the association of weight loss modalities to body contouring outcomes emphasizing b and drainage fluid volumes. It focuses on how bariatric surgery affects perioperative hemoglobin and anemia levels as well as drainage fluid volumes in abdominoplasty patients. To our knowledge, this is the first study investigating the effects of different massive weight loss forms on postoperative fluid changes.

## 2. Materials and Methods

### 2.1. Study Design and Patient Analysis

In this study, we analyzed patients undergoing abdominoplasty at the Clinical Department for Plastic, Aesthetic and Reconstructive Surgery at the University Hospital St. Poelten between 1 January 2018 and 31 December 2023. This study was conducted as a retrospective single-center study. Ethical approval was obtained from the local institutional review board at the Karl Landsteiner University of Health Sciences Krems (reference number: ECS 1048/2024). Analyzed factors included the patients’ age at surgery, BMI, resection weight, perioperative hemoglobin levels, postoperative drainage fluid volume, duration of surgery and duration of hospital stay.

Given the critical role of hemoglobin in oxygen transport and organ function, this study specifically examined hemoglobin levels following body contouring surgery in both bariatric and non-bariatric patients, as well as changes in drainage fluid volumes. Since the underlying causes of anemia were not the primary focus, parameters such as MCH, MCV and MCHC were not extensively analyzed. Hemoglobin values (g/dL) were analyzed between 1 to 7 days prior to surgery and on the first postoperative day. Anemia was disclosed as values below 12 g/dL according to the WHO classification [15]. Drainage output was documented every 12 h until the removal of the drainage catheters. Drainage removal was conducted if the output was less than 30 mL in 24 h.

### 2.2. Inclusion and Exclusion Criteria 

In our study, we enrolled male and female patients aged between 18 and 75 years who had experienced massive weight loss and demonstrated stable weight maintenance for a minimum duration of one year. Eligibility required the presence of significant redundant skin localized to the abdominal region. Participants were included if they had achieved a weight reduction corresponding to at least 70% of their initial excess weight. The patients’ maximum pre-weight loss body weight was verified either by their general practitioner or by a specialist in plastic surgery within our department. To further ensure weight stability, patient weight was re-assessed immediately prior to surgery. Only individuals who underwent conventional abdominoplasty were considered for inclusion; patients who received a fleur-de-lis abdominoplasty were excluded, as this more extensive technique is associated with a higher incidence of wound healing complications, which could confound drainage volume measurements. With respect to the method of weight loss, we included both patients who had undergone bariatric surgical procedures—such as gastric bypass, gastric banding or sleeve gastrectomy, including any combination thereof—and those who achieved weight loss through lifestyle modification. Only patients with preoperative hemoglobin levels of at least 11 mg/dL and a hematocrit of at least 33% were eligible for inclusion.

Exclusion criteria were rigorously applied to minimize confounding variables. Patients who had been treated with oral antidiabetic medications, such as Ozempic^®^, were excluded from the study. Additionally, individuals who required postoperative revision surgery due to anemia requiring blood transfusions or clinically significant bleeding were not included, as such interventions could alter the analysis of the drainage volumes. None of the patients included in our cohort underwent revision surgery in the abdominal region. We further excluded patients with hematopoietic disorders, bone marrow abnormalities, malignancies or other similar conditions that could affect wound healing or drainage. Patients undergoing combined surgical procedures, such as gluteal or thigh lifts, were also excluded, as the creation of a contiguous wound cavity between the abdominal and adjacent regions could compromise the accuracy of drainage measurements. Lastly, individuals receiving anticoagulation therapy (e.g., for atrial fibrillation) were not eligible for participation.

### 2.3. Operative Procedure

Abdominoplasty at our department was performed conventionally with a horizontal skin incision starting approximately 6–8 cm above the anterior commissure with the skin stretched. Preparation then proceeded down to the abdominal fascia. Dissection was then performed cranially until reaching the umbilicus, which was precisely prepared. Further dissection was conducted only centrally until reaching the xyphoid. After the pannus resection was performed, closure was carried out by suturing the scarpa’s fascia, the corium and the skin. One redon drainage per side was installed and exited outside or through the wound. If needed, a rectus lift was performed with continuous or interrupted sutures. Patients received abdominal bandages after skin closure and plaster bandages. Compression garments were installed immediately after surgery and worn for 6–12 weeks for 24 h.

### 2.4. Statistics and Data Management

The endpoints of our analyses were to assess hemoglobin loss and the volume of postoperative drainage fluids after body contouring surgeries. Patients were divided into two groups: patients experiencing abdominoplasty without and with bariatric surgery beforehand. All data were anonymized prior to analysis, and data protection management adhered to Austrian legal requirements. Data collection and processing were conducted using Microsoft Excel (Microsoft Corp., Redmond 98052-6399, Washington, TX, USA), while statistical analyses were performed with IBM SPSS Statistics version 29 (IBM, Armonk, NY, USA). Nominal data are presented as absolute frequencies and percentages; metric data are reported as means and standard deviations. Paired *t*-tests were used to compare postoperative drainage volumes and hemoglobin values between patients. To assess the relationship between postoperative drainage fluid, hemoglobin loss and bariatric procedures, Spearman Rho correlation analyses were performed. Further analyses employing the Mann–Whitney U test were conducted to perform accurate correlation analyses while accounting for outliers. Two-sided *p* ≤ 0.05 was regarded as statistically significant.

## 3. Results

In total, 505 body forming procedures were analyzed in this study. Of these, three were excluded due to postoperative revision of bleeding complications, six were excluded because of solely being extensive scar revision surgeries and sixty-one had to be excluded due to data insufficiency. Of 435 body contouring surgeries, 184 were thigh lift procedures and 47 were gluteal lift surgeries. These 231 procedures were excluded due to inconsistent datasets and the possible falsification of drainage volumes (especially in the case of a combined gluteal lift). Finally, 204 abdominoplasties in massive weight loss patients met our criteria and were included in this study. Here, 80 abdominoplasties were performed in patients experiencing weight loss due to lifestyle changes and 124 procedures were performed in bariatric patients.

### 3.1. Modality of Weight Loss

Patients included in our analyses underwent weight loss either due to lifestyle changes or bariatric surgery. Lifestyle changes included dietary adjustments, caloric deficit and exercise (either endurance or strength training or both). Patients who underwent a bariatric procedure mainly had gastric bypass surgery (111, 89.51%). A sleeve gastrectomy procedure was the second most common intervention (9, 7.26%). Only a few patients underwent gastric band procedure (4, 3.23%). All bariatric procedures were performed at external centers. Patients who underwent bariatric surgery maintained their weight for at least 6–12 months prior to body contouring at our department. Patients who lost weight through lifestyle changes maintained their weight for at least 12 months before undergoing body contouring surgery.

### 3.2. Patient Demographics

In total, 124 patients underwent bariatric surgery, whereas 80 patients achieved weight loss through lifestyle changes (Table 1). The mean age at surgery was 42.82 years ± 11.33 years for lifestyle patients and 42.87 years ± 10.98 years for bariatric patients. The mean BMI following weight loss through lifestyle changes was 26.17 kg/m^2^ ± 4.58 kg/m^2^ and 26.33 kg/m^2^ ± 3.72 kg/m^2^ through bariatric surgery, respectively. Mean preoperative Hb values were 14.02 g/dL ± 1.22 g/dL in lifestyle patients and 13.24 g/dL ± 1.38 g/dL in bariatric patients. Postoperatively, patients showed a mean Hb of 11.71 g/dL ± 1.59 g/dL and 10.68 g/dL ± 1.48 g/dL. Resection weight in lifestyle patients and bariatric patients was 1364.90 g ± 1108.29 g and 1442.28 g ± 1359.51 g. The duration of surgery was 179.43 min ± 89.86 min in lifestyle patients and 213.89 min ± 87.26 min in bariatric patients. The duration of surgery was longer in the case of teaching cases. Regarding hospital stay, lifestyle patients showed a mean of 6.49 days ± 2.68 days and bariatric patients a mean of 6.74 days ± 2.40 days. The postoperative drainage volume was 342.21 mL ± 429.77 mL in lifestyle patients and 442.38 mL ± 405.42 mL in bariatric patients. The drainage inlay time was 4.78 days ± 2.42 days and 5.32 days ± 2.38 days.

### 3.3. Hemoglobin Alternation

**Figure 1:**
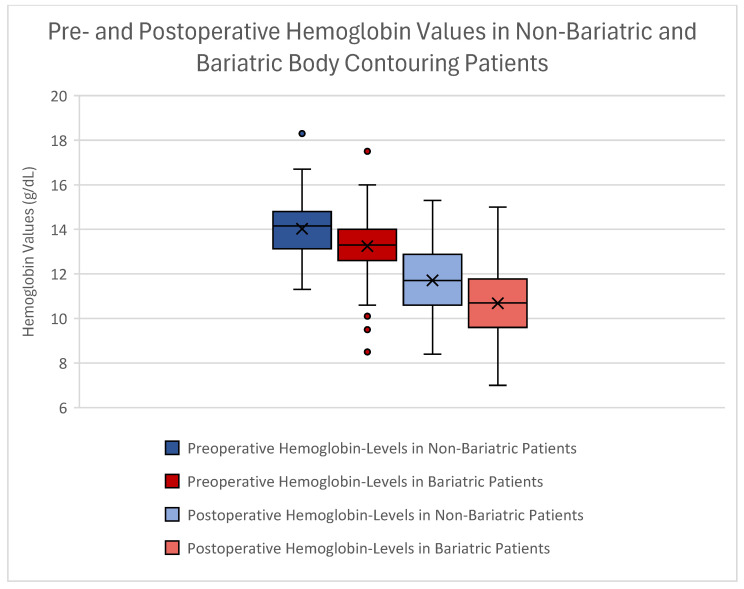
Boxplot of Hb values comparing patients with abdominoplasty after lifestyle changes (blue) and bariatric surgery (red). The significant difference between pre- and postoperative Hb values can be seen within this graph. Preoperative Hb values are displayed in dark blue (lifestyle changes) and dark red (bariatric surgery). Postoperative Hb values are seen in light blue (lifestyle changes) and light red (bariatric surgery). The x marks mean values. Outliers can be seen as dots.

Patients without bariatric surgery showed preoperative Hb values of 14.02 g/dL ± 1.22 g/dL, whereas patients with bariatric surgery showed mean preoperative Hb values of 13.24 g/dL ± 1.38 g/dL (Figure 1). Postoperatively, lifestyle abdominoplasty patients showed a mean Hb value of 11.71 g/dL ± 1.59 g/dL. Contrarily, bariatric patients showed mean Hb levels of 10.68 g/dL ± 1.48 g/dL.

Using an independent sample *t*-test, we found a significant difference in both preoperative and postoperative hemoglobin levels between the groups (*p* < 0.001 for both). However, the extent of hemoglobin loss did not differ significantly between non-bariatric and bariatric patients (*p* = 0.246) (see Table 2).

**Figure 2:**
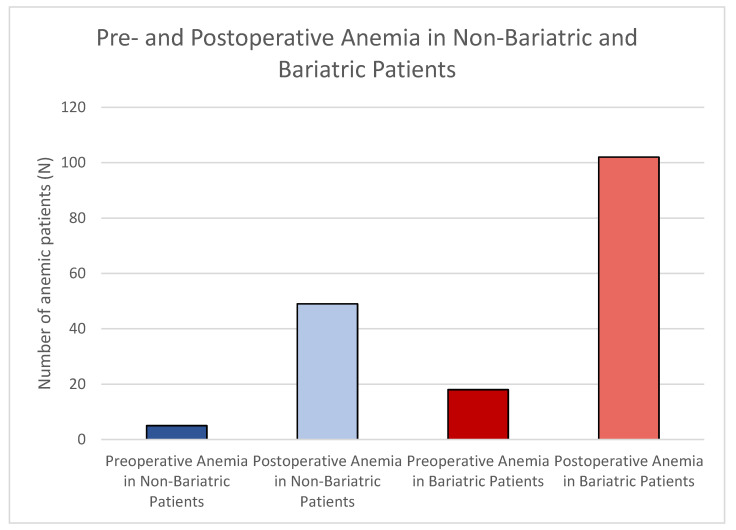
Bar diagram of pre- and postoperative anemia among abdominoplasty patients. Preoperative anemia in lifestyle patients can be seen in dark blue; postoperative anemia among lifestyle patients is depicted in light blue. Preoperative anemia in bariatric patients can be seen in dark red; postoperative anemia can be seen in light red. These differences were statistically significant preoperatively and postoperatively.

Lifestyle patients were anemic preoperatively in 5 (6.25%) cases and postoperatively in 49 (61.25%) cases. Bariatric patients showed anemia in 18 (14.52%) cases preoperatively and in 102 (82.26%) cases postoperatively (Figure 2).

By conducting a *t*-test for Equality of Means, we observed that there was a significant difference in preoperative anemia between our groups (*p* = 0.05). Additionally, a significant difference in postoperative anemia between our groups was also seen (*p* = 0.001) (Table 3).

We observed that bariatric surgery significantly affected hemoglobin levels both preoperatively (*p* < 0.001) and postoperatively (*p* < 0.001), but not intraoperative hemoglobin loss (*p* = 0.201). Additionally, there was a significant correlation with postoperative anemia and bariatric surgery (*p* < 0.001), although not between preoperative anemia and bariatric surgery (*p* = 0.069).

### 3.4. Drainage Fluid Volume and Drainage Inlay Time

**Figure 3:**
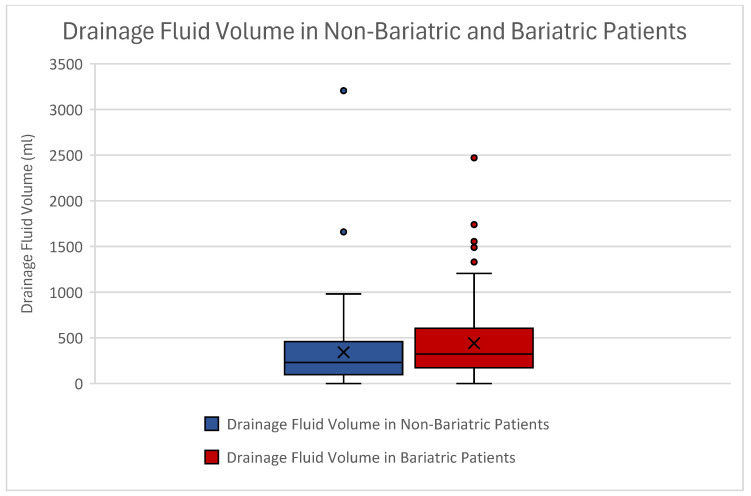
Boxplot of drainage fluid volume among abdominoplasty patients without (blue) and with (red) bariatric surgery. Drainage volumes differed significantly between our groups when we conducted the Mann–Whitney U test. The x marks mean values. Outliers can be seen as dots.

Patients without bariatric surgery had a mean drainage fluid volume of 342.21 mL ± 429.77 mL. Those with bariatric procedures showed a mean volume of 442.38 mL ± 405.42 mL (Figure 3).

After performing non-parametric analyses by conducting the Mann–Whitney U test, a statistical significance in drainage volume could be seen between our groups (*p* = 0.01).

By correlating bariatric surgeries to the postoperative drainage fluid volume, a direct association was identified between the two (*p* = 0.009) (Table 4). This proves that bariatric surgeries increased postoperative drainage fluid output. Further, the postoperative drainage volume was significantly increased in the case of postoperative anemia (*p* = 0.024), lower postoperative Hb values (*p* < 0.039) and increased resection weight (*p* < 0.001). Nonetheless, no significant difference regarding resection weight was identified between our groups (*p* = 0.671).

**Figure 4:**
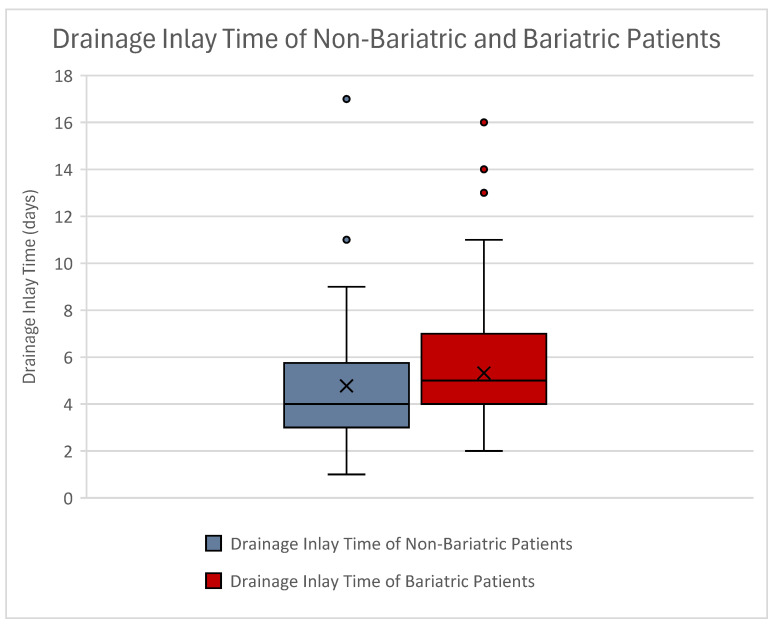
Boxplot of drainage inlay time in non-bariatric (blue) and bariatric patients (red). Note that drainage inlay time was higher in bariatric patients. Still, this finding proved not to be statistically significant. Outliers can be seen as dots.

The mean drainage inlay time was 4.78 days ± 2.42 days in lifestyle-associated weight loss patients and 5.32 days ± 2.38 days in bariatric weight loss patients. After performing an independent sample test using the Mann–Whitney U test, no significant difference could be observed when investigating drainage inlay time (*p* = 0.081). Spearman Rho analyses also showed no direct correlation of bariatric surgeries to drainage inlay time (*p* = 0.081). Drainage inlay time only significantly correlated to the duration of surgery (*p* = 0.007) and resection weight (*p* < 0.001) (Figure 4).

### 3.5. Duration of Surgery and Hospital Stay

**Figure 5:**
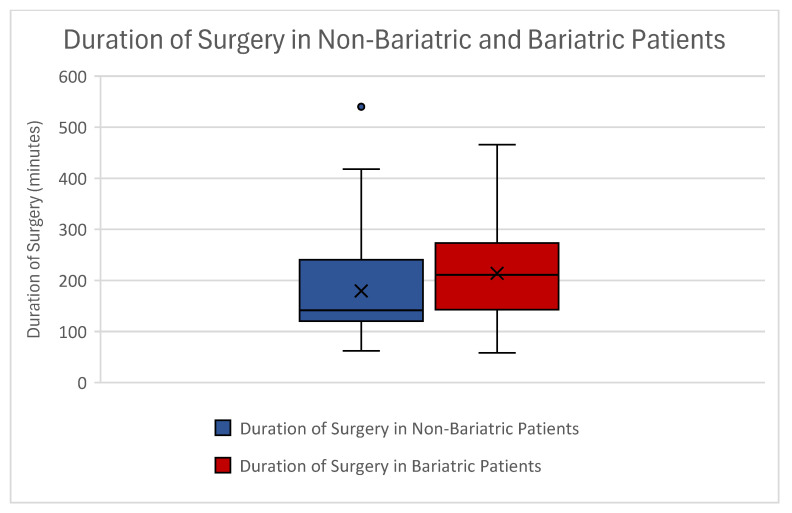
Boxplot showing duration of surgery between our patients. Non-bariatric patients can be seen in blue and bariatric patients can be seen in red. This finding showed a significant difference. Outliers can be seen as dots.

Patients undergoing abdominoplasty after weight loss due to lifestyle changes had a mean duration of surgery of 179.43 min ± 89.86 min. Bariatric abdominoplasty patients had a mean duration of surgery of 213.89 min ± 87.26 min (Figure 5).

An independent *t*-test showed a significant difference in the duration of surgery between both groups (*p* = 0.007) (Table 5).

After performing correlation analyses, we further observed that bariatric procedures significantly correlated with a longer duration of surgery (*p* = 0.002). In addition, an increased duration of surgery correlated with increased hemoglobin loss (*p* < 0.001). This might be due to the alternating surgical team and training assignments at our department to properly educate young plastic surgeons.

**Figure 6:**
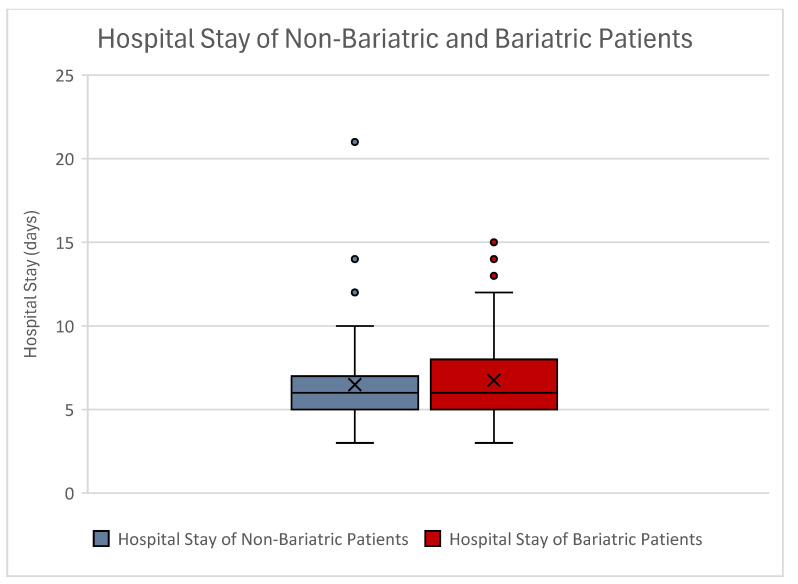
Boxplot of hospital stay between non-bariatric (blue) and bariatric (red) patients. No statistical significance was seen between both groups. Outliers can be seen as dots.

The mean hospital stay in non-bariatric abdominoplasty patients was 6.49 days ± 2.68 days, whereas bariatric patients had a mean hospital stay of 6.74 days ± 2.40 days. Our correlation analyses using the *t*-test showed no statistical significance regarding hospital stay (*p* = 0.481). Also, Spearman Rho analyses showed no correlation of bariatric surgery to extended hospital stay (*p* = 0.245) (Figure 6).

## 4. Discussion

More than one billion people worldwide suffered from obesity in 2022, accounting for an estimate of 12.5% of the global population [16]. This trend is consistent with recent analyses showing a near tripling of obesity rates since 1990, particularly in high-income countries. While lifestyle changes can be highly effective for weight reduction, bariatric surgery remains pivotal for severe cases [17,19,20,21]. Because of the high success of massive weight loss, patients are often left with severe skin laxity resulting in grave distress [13,22,23]. Body contouring procedures, particularly abdominoplasty, are beneficial by removing excess skin and improving functionality and appearance [4,13,14,22,23,24,25,26,27,28,29,30]. Beyond the high frequency of performed abdominoplasties worldwide, affiliated complications are not to be neglected [31]. This study focuses on the concordant perioperative fluid balance of non-bariatric and bariatric patients experiencing abdominal body contouring.

Patients who have undergone bariatric surgery and achieved significant weight reduction frequently encounter a spectrum of postoperative challenges, most notably malabsorption, digestive disturbances and a range of nutritional deficiencies [32,33,34,35,36,37,38]. Notably, the absorption of iron, vitamin B12 and folic acid is restricted [39,40,41,42,43]. This could already be stated by previous studies showing that such deficiencies commonly lead to impaired hematopoiesis and increased rates of anemia [44,45,46]. The anatomical changes following procedures such as gastric bypass directly compromise the absorption of these nutrients, as evidenced by reduced transport across the mucosal barrier.

Our data show significantly lower pre- and postoperative hemoglobin (*p* < 0.01] levels in bariatric patients, with a corresponding higher incidence of anemia (*p* = 0.001), supporting earlier reports of the hematologic challenges faced by this population. Correlation analyses further substantiated these associations (p_all_ < 0.01). The underlying cause of these observations is likely related to the preexisting absorption deficiencies frequently observed in individuals following bariatric surgery [47,48,49,50,51].

Our findings on fluid management both reinforce and expand upon the existing literature. Drainage volumes differed significantly between groups (*p* = 0.01), with bariatric patients exhibiting higher outputs (*p* = 0.009), particularly among those with lower postoperative hemoglobin levels (*p* = 0.039). These results are consistent with the work of Shermak et al. who reported increased seroma formation and drainage output in post-bariatric patients undergoing abdominoplasty [52]. The underlying pathophysiology likely involves impaired hemostasis and reduced clotting capacity in anemic individuals, which compromise effective coagulation and increase postoperative fluid loss. Moreover, diminished tissue oxygenation and delayed wound healing—both sequelae of anemia—may further exacerbate drainage output. Anemic patients also tend to receive greater volumes of intravenous fluids, potentially diluting clotting factors and prolonging drainage, as previously described by Ling Li et al. [53].

While the duration of drainage inlay was not directly associated with bariatric surgery (*p* = 0.081) in our study, it did correlate with longer operative times (*p* = 0.007), which themselves were linked to both bariatric procedures (*p* = 0.002) and postoperative anemia (*p* < 0.001). This may reflect increased tissue trauma, leading to greater capillary and lymphatic disruption, and, consequently, more fluid leakage. In post-bariatric body contouring, larger tissue areas are often exposed, potentially increasing desiccation and inflammatory responses, and thereby promoting serous exudation as already reported by Kitzinger et al. [54]. Although a direct correlation between bariatric history and length of hospital stay was not observed (*p* = 0.245), prolonged hospitalization was associated with lower postoperative hemoglobin levels (*p* < 0.001) and a higher incidence of anemia (*p* = 0.005), both of which were more prevalent in post-bariatric patients. This may be due to delayed wound healing and reduced mobilization in this group.

Our study provides evidence that the hematologic consequences of bariatric surgery—most notably chronic anemia due to the persistent malabsorption of iron and vitamin B12—have a direct and clinically significant impact on postoperative outcomes in body contouring surgery. The observed association between lower pre- and postoperative hemoglobin levels and increased drainage volumes suggests that impaired hemostasis and reduced oxygen-carrying capacity in bariatric patients may compromise wound healing and fluid homeostasis.

The pathophysiology underlying these observations is multifactorial. Anemia impairs tissue oxygenation, which is essential for effective wound healing and the formation of stable fibrin clots. Inadequate clot formation can lead to persistent oozing from the surgical site, thereby increasing drainage volumes. Furthermore, iron and vitamin B12 deficiencies can negatively affect the immune response, potentially delaying the resolution of inflammation and further prolonging fluid exudation. The tendency for anemic patients to receive greater volumes of intravenous fluids intra- and postoperatively may also contribute to dilutional coagulopathy, compounding the risk of excessive drainage.

These insights underscore the importance of comprehensive preoperative assessment and optimization in post-bariatric patients scheduled for body contouring procedures. Routine screening for iron, vitamin B12 and folic acid deficiencies should be standard practice, with aggressive correction of any deficits prior to surgery. This approach is supported by recent guidelines from the American Society for Metabolic and Bariatric Surgery, which advocate for lifelong nutritional surveillance in this patient group [55]. Additionally, perioperative management protocols should be tailored to minimize unnecessary fluid administration and to monitor for signs of impaired coagulation.

Given the heterogeneity of post-bariatric patients, individualized perioperative care plans may also be warranted. Bariatric patients with documented anemia or borderline hematologic indices may benefit from addressing nutritional deficiencies preoperatively. Further, preoperative iron supplementation or erythropoiesis-stimulating agents may also be considered. Intraoperatively, meticulous hemostasis and judicious fluid management are paramount. Fluid management protocols might aid in reducing excessive drainage and its associated complications. Postoperatively, early mobilization and close monitoring of wound healing and drainage output can further help identify complications promptly, allowing for timely intervention. These observations invite a broader consideration of the complex interplay between hematologic status and fluid management in post-bariatric body contouring patients.

Our results also have extensive implications for the management of other surgical populations with similar risk profiles, such as patients with chronic gastrointestinal disorders or those undergoing major reconstructive procedures. The interplay between nutritional status, hematologic health and surgical outcomes is likely relevant across a range of clinical contexts. The association between anemia, impaired hemostasis and increased drainage output suggests that perioperative care for this population may benefit from a more nuanced approach.

Moreover, the indirect relationship between anemia and prolonged hospital stay raises questions about the downstream effects of altered wound healing and mobilization in post-bariatric patients. It is plausible that these factors, influenced by gastrointestinal changes after bariatric surgery, contribute to a more complex recovery trajectory. This underscores the need for interdisciplinary collaboration among surgeons, nutritionists and anesthesiologists to optimize outcomes.

## 5. Limitations

Despite the valuable insights provided by our study, several limitations must be acknowledged. The sample distribution and variability in bariatric procedures may have introduced selection bias and affected generalizability. It also might have limited statistical analyses. This heterogeneity complicates the extrapolation of our findings to broader patient populations. Moreover, the variability in bariatric techniques employed across the cohort presented an additional challenge. Each surgical method—whether gastric bypass, sleeve gastrectomy or adjustable gastric banding—had unique metabolic and nutritional consequences. These differences can influence not only the rate and extent of weight loss but also the patient’s postoperative nutritional status, wound healing capacity and risk of complications. As a result, outcome inconsistencies may arise, making it difficult to draw definitive conclusions about the relationship between bariatric history and the success of body contouring procedures.

Another limitation was in the retrospective nature of our study design, which is inherently subject to information bias and incomplete data collection. The lack of standardized follow-up intervals and assessment tools may further contribute to variability in outcome reporting. Additionally, confounding factors such as patient comorbidities, adherence to nutritional supplementation and differences in perioperative care protocols were not fully controlled for, which could have influenced the observed results.

Given these constraints, our findings should be interpreted with caution. To overcome these limitations and strengthen the evidence base, future research should focus on prospective studies with larger, more homogeneous patient cohorts. The implementation of standardized surgical and follow-up protocols would enable more precise comparisons and facilitate meta-analyses. Ultimately, such efforts are essential to deepen our understanding of how different bariatric histories impact the outcomes of body contouring surgery, and to optimize patient care in this growing field.

## 6. Conclusions

Our findings highlight the particular challenges encountered by post-bariatric patients undergoing body contouring procedures, especially regarding perioperative anemia and increased drainage requirements. These associations suggest the importance of individualized hemoglobin management strategies—such as tailored nutritional interventions—for post-bariatric patients, with the aim of improving postoperative care and potentially minimizing hemoglobin loss. As the number of post-bariatric body contouring procedures continues to rise, a deeper understanding of these factors will be crucial for optimizing patient outcomes and enhancing safety. Future prospective studies are warranted to further elucidate these relationships and inform best practices.

## Figures and Tables

**Table 1:** Demography of abdominoplasty patients with weight reduction due to lifestyle changes (left) and bariatric surgery (right) included in this study.

Patient Characteristics		Abdominoplasty—Lifestyle Change	Abdominoplasty—Bariatric Surgery
Number		80	124
Gender	F	64 (80%)	113 (91.13%)
	M	16 (20%)	11 (8.87%)
Age (years)	Mean	42.82	42.87
	STD	±11.33	±10.98
BMI (kg/m^2^)	Mean	26.17	26.33
	STD	±4.58	±3.72
Hb pre-op (g/dL)	Mean	14.02	13.24
	STD	±1.22	±1.38
Hb post-op (g/dL)	Mean	11.71	10.68
	STD	±1.59	±1.48
Hb difference pre-post	Mean	−2.30	−2.56
	STD	±1.43	±1.62
Anemia pre-op	Yes	5 (6.25%)	18 (14.52%)
	No	75 (93.75%)	106 (85.48%)
Anemia post-op	Yes	49 (61.25%)	102 (82.26%)
	No	31 (38.75%)	22 (17.74%)
Resection weight (g)	Mean	1364.90	1442.28
	STD	±1108.29	±1359.51
Duration of surgery (min)	Mean	179.43	213.89
	STD	±89.86	±87.26
Hospital stay (days)	Mean	6.49	6.74
	STD	±2.68	±2.40
Drainage volume (mL)	Mean	342.21	442.38
	STD	±429.77	±405.42
Drainage inlay time (days)	Mean	4.78	5.32
	STD	±2.42	±2.38

**Table 2:** *T*-test for Equality of Means showing a significant difference in pre- and postoperative Hb values in both groups after abdominoplasty (*p* < 0.001 pre- and postoperative). Nonetheless, there was no significance in Hb loss seen between our groups (*p* = 0.246).

*T*-Test for Equality of Means							
	F	Sig.	T	df	One-Sided *p*	Two-Sided *p*	Mean Difference
Hemoglobin preoperative	0.486	0.486	4.127	202	<0.001	<0.001	0.7818
Hemoglobin postoperative	0.678	0.411	4.678	202	<0.001	<0.001	1.0253
Hemoglobin difference	1.227	0.269	−1.164	202	0.123	0.246	−0.258

**Table 3:** *T*-test for Equality of Means showing a significant difference in pre- and postoperative anemia between patients without, versus with, bariatric surgery beforehand (preoperative = 0.05 and postoperative = 0.001).

*T*-Test for Equality of Means							
	F	Sig.	T	df	One-Sided *p*	Two-Sided *p*	Mean Difference
Anemia preoperative	14.865	<0.001	−1.976	201.102	0.025	0.05	−0.082
Anemia postoperative	38.646	<0.001	−3.245	139.724	<0.001	0.001	−0.2100

**Table 4:** Spearman Rho correlation analysis demonstrating that bariatric procedures do influence the amount of drainage fluid volume (*p* = 0.009). Further, drainage volume significantly correlated to low postoperative Hb values (*p* = 0.039), postoperative anemia (*p* = 0.024) and resection weight (*p* < 0.001).

Spearman Rho Correlation Analysis			
Spearman Rho	Drainage Volume	Corr. Coefficient	1.000
		Sig. (2-tailed)	.
		N	204
	Bariatric surgery	Corr. Coefficient	0.182
		Sig. (2-tailed)	0.009
		N	204
	Hemoglobin postoperative	Corr. Coefficient	−0.145
		Sig. (2-tailed)	0.039
		N	204
	Anemia postoperative	Corr. Coefficient	0.158
		Sig. (2-tailed)	0.024
		N	204
	Resection weight	Corr. Coefficient	0.247
		Sig. (2-tailed)	<0.001
		N	204

**Table 5:** *T*-test for Equality of Means showing a significant difference in duration of surgery between non-bariatric and bariatric abdominoplasty patients (*p* = 0.008).

*T*-Test for Equality of Means							
	F	Sig.	T	df	One-Sided *p*	Two-Sided *p*	Mean Difference
Duration of Surgery	0.017	0.897	−2.722	202	0.004	0.007	−34.4621

## Data Availability

All the data analyzed during the current study are available from the corresponding author on reasonable request.
